# Trends in Hospitalization for Fall-Related Injuries in US Older Adults, 2001–2021

**DOI:** 10.1155/tswj/8340466

**Published:** 2025-04-19

**Authors:** Carlos H. Orces

**Affiliations:** Department of Medicine, Laredo Medical Center, Laredo, Texas, USA

## Abstract

**Introduction:** Fall-related injuries are a major public health problem affecting ageing populations. Although previous studies have reported increasing hospitalization rates for fall-related injuries in United States older adults, recent trends in hospitalization for these injuries have not been examined. Thus, the present study aimed to examine nationwide trends in hospitalization after emergency department (ED) visits for fall-related injuries.

**Methods:** The National Electronic Injury Surveillance System-All Injury Program was used to estimate hospitalizations after ED visits for fall-related injuries in adults ≥ 65 years between 2001 and 2021. Joinpoint regression software V.5.0.2 was used to examine the average annual percent change in age- and sex-adjusted hospitalization rates according to type of injury, body parts, and traumatic brain injuries.

**Results:** A total of 704,875 ED visits for fall-related injuries occurred in US older adults between 2001 and 2021. Of those 26.5% (95% CI: 26.3, 26.6) were hospitalized. Overall, women represented 59.2% (95% CI: 59.0, 59.5) of the hospitalizations and fractures were the leading type of injury. By sex, hospitalization rates in women annually increased on average by 2.5% (95% CI: 2.0, 3.0) and in men by 4.2% (95% CI: 3.6, 4.9). Similarly, fall-related fracture rates in men increased annually on average by 2.5% (95% CI: 1.9, 3.2) and in women by 1.5% (95% CI: 1.0, 2.2) during the study period. Notably, the highest increase in hospitalization rates during the study period occurred among older adults with traumatic brain injuries.

**Conclusion:** Hospitalizations for fall-related injuries significantly increased in US older adults over the past two decades. The present findings underscore the importance of implementing effective community-based programs to prevent fall-related injuries.

## 1. Introduction

Fall-related injuries in older adults represent a public health burden, often resulting in emergency department (ED) visits and hospitalizations [1]. About one-third of ED visits for fall-related injuries in older adults require hospitalization. Of those, fall-related fractures and internal injuries account for the leading diagnoses [2]. Previous studies have demonstrated that the incidence of hospitalizations after ED visits for fall-related injury in older adults increased on average by 3.3% and 4% per year during the periods 2001–2008 and 2001–2012, respectively [1, 3]. Nevertheless, recent trends in hospitalization for fall-related injuries among older adults have not been examined. Thus, the present study aimed to examine trends in hospitalization rates for fall-related injuries among US older adults between 2001 and 2021.

## 2. Materials and Methods

The National Electronic Injury Surveillance System-All Injury Program (NEISS-AIP) was used to estimate hospital admissions after ED visits for fall-related injuries in US adults aged ≥ 65 years. The NEISS-AIP collects nonfatal injuries in a stratified probability sample of approximately 100 hospitals that have at least 6 beds and provide 24-h ED services. Trained coders abstracted data for injury-related cases from electronic ED medical records. Data obtained on each case include age, race/ethnicity, gender, principal diagnosis, primary body part affected, primary–secondary body part-grouped, disposition at ED discharge, place where the injury occurred, and a narrative description of the injury circumstances. Moreover, the data included major categories of external cause of injury (e.g., motor vehicle, falls, cut/pierce, poisoning, and fire/burn) and of intent of injury [4].

Age-adjusted hospitalization rates per 100,000 population stratified by sex and according to type of injury, affected body parts, and traumatic brain injuries (TBIs) were calculated by the direct method using the US 2000 population as the standard. TBIs in the NEISS-AIP data were defined as a head injury (primary body part) combined with an injury diagnosis of internal injury or concussion [2].

### 2.1. Statistical Analysis

Joinpoint trend analysis software V.5.0.2 was used to report the average annual percent change (AAPC) in hospitalization rates between 2001 and 2021. This summary measure describes the average annual percent changes during a period, which is valid even if the regression model indicates changes in trends over time. In addition, the annual percent change (APC) in rates was reported if a significant change in trend was identified during the study period. A maximum of 2 joinpoints and a minimum of 6 observation between 2 jointpoints were allowed for each analysis. A detailed description of the methods used in the Joinpoint trend analysis can be found at: https://surveillance.cancer.gov/joinpoint/tutorials.html. Stata 18 (Stata Corp, College Station, TX) was used to incorporate NEISS-AIP sampling weights and compute national estimates of hospitalization for fall-related injuries.

## 3. Results

A total of 704,875 ED visits for fall-related injuries occurred in US older adults between 2001 and 2021. Of those 26.5% (95% CI: 26.3, 26.6) required hospital admission. Overall, 66.4% (95% CI: 66.2, 66.7) of hospitalizations occurred in women and fall-related fractures accounted for 59.2% (95% CI: 59.0, 59.5) of the injuries. Notably, the percentage of older adults hospitalized for fall-related injuries increased from 20.7% (95% CI: 20.1, 21.3) in 2001 to 30.9% (95% CI: 30.5, 31.4) in 2021.

**Figure 1:**
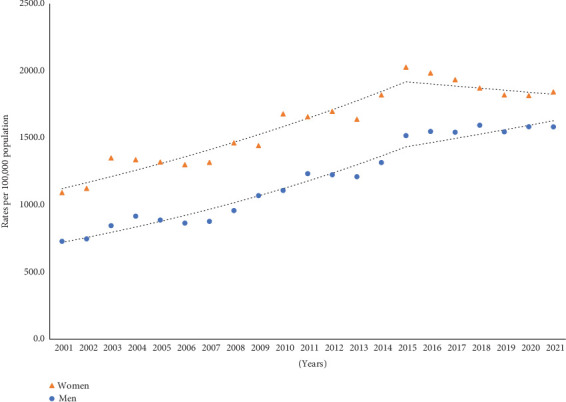
Trends in fall-related hospitalization rates in older adults, 2001–2021.

Age-adjusted fall-related hospitalization rates were higher in women than those in men. In women, hospitalization rates increased on average by 2.5% (95% CI: 2.0, 3.0) per year. Although the rates among women annually increased by 3.9% (95% CI: 3.4, 5.0) between 2001 and 2015, a nonsignificant decline in rates was seen from 2015 onward. In men, hospitalization rates gradually increased on average by 4.2% (95% CI: 3.6, 4.9) per year (Figure 1).

As shown in Table 1, fall-related fractures accounted for the majority of hospital admissions. In men, fall-related fracture rates gradually increased by 2.5% (95% CI: 1.9, 3.2) per year. Similarly, the rates in women increased annually on average by 1.5% (95% CI: 1.0, 2.2) between 2001 and 2021. However, a nonsignificant decline in fracture rates was seen between 2015 and 2021. Of note, the AAPC in hospitalization rates for internal injuries markedly increased in men by 10% (95% CI: 9.1, 11.2) and in women by 8.8% (95% CI: 7.6, 10.2).

Lower trunk and head/neck injuries were the leading cause of hospitalizations in women and men, representing 42.6% (95% CI: 42.0%, 47.2%) and 39.3% (CI: 38.5%, 39.4%), respectively. As shown in Table 2, hospitalization rates for lower trunk injuries in women annually increased by 1.6% (95% CI: 0.9%, 3.2%) between 2001 and 2015. Subsequently, the rates significantly decreased by −4.0% (95% CI: −6.0%, −1.1%) per year between 2015 and 2021. Similarly, hospitalization rates for lower trunk injuries decreased in men by −3.1% (95% CI: −5.3%, −0.4%) per year during 2015–2021. In contrast, the AAPC in hospitalization rates for head/neck injuries in men annually increased by 7.1% (95% CI: 6.4%, 8.0%) and in women by 5.8% (95% CI: 4.8%, 6.9%).

**Figure 2:**
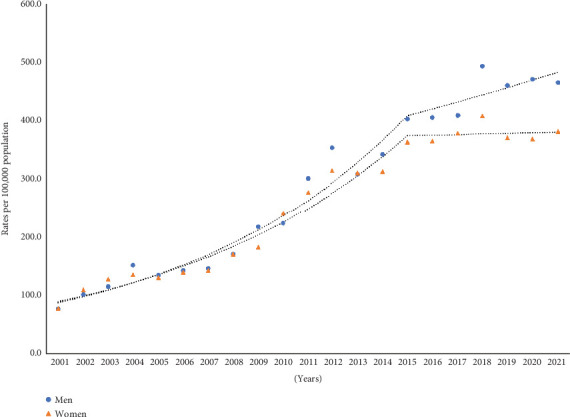
Trends in hospitalizations for fall-related traumatic brain injuries, 2001–2021.

As shown in Figure 2, the AAPC in hospitalization rates for TBIs significantly increased by 8.9% (95% CI: 8.0%, 10.1%) in men and by 7.5% (95% CI: 6.5%, 8.6%) in women between 2001and 2021. However, a nonsignificant downward trend in TBI-related hospitalizations in women by 0.3% (95% CI: −3.0%, 4.7%) per year was seen from 2015 onward. In men, fall-related TBI hospitalization rates continued to increase throughout the study period.

## 4. Discussion

The present findings indicate that hospitalization rates for fall-related injuries admitted through the ED increased in US older adults between 2001 and 2021. Upward trends in hospitalizations were predominantly attributed to an increase in the incidence of fall-related fractures, internal injuries, head/neck injuries, and TBIs. Notably, hospitalization rates for lower trunk injuries significantly decreased in men and women from 2015 onward. Likewise, among older women, a nonsignificant downward trend in hospitalization rates for fall-related fractures and TBIs occurred during 2015–2021.

Consistent with the present findings, prior studies reported increasing hospitalization rates for fall-related injuries in US older adults over the past decade [1, 3]. Similarly, upward trends in hospitalizations for fall-related injuries were demonstrated in European countries [5–7]. Taylor et al. using data from the National Inpatient Sample similarly reported that fall-related TBI hospitalization rates increased by 37% among adults aged 75 years and older between 2007 and 2013 [8]. Likewise, a recent report demonstrated that hospitalization rates after ED visits for fall-related head/neck fractures in older adults increased on average by 3.1% per year during 2001–2020 [9]. The marked increase in hospitalizations for TBIs in the USA has been partly explained owing the introduction of the National Center for Injury Prevention and Control 2010 revised guidelines for the diagnosis and management of head injuries, which have resulted in more public awareness about the effects of TBIs and medical care [10].

Hospitalization rates for lower trunk injuries remained steady during the study period. However, a significant decrease in hospitalization rates for these injuries occurred in men and women from 2015 onward. The present findings are consistent with those from a recent report in which hospitalization rates for fall-related lower trunk fractures did not significantly change in US older adults between 2001 and 2020, but a nonsignificant decrease in ED visits for fall-related fractures among older adults occurred between 2016 and 2020 [8].

Lewiecki et al. also reported that the incidence of hip and pelvis fractures decreased by 33% and 49% among US commercial and Medicare Advantage health plan members aged ≥ 50 years between 2007 and 2017, respectively [11]. Similarly, Reider et al. demonstrated that nationwide hospitalization rates for low-energy femoral fractures in older adults decreased by 20% between 2003 and 2017 [12]. The study results also indicate that a nonsignificant recent decline in hospitalization for fall-related fractures among older women occurred between 2015 and 2021. Although explanation for this finding is unknown, trends in osteoporosis medication use among US postmenopausal women decreased over the past decade and its use was suboptimal even in those with prevalent osteoporosis [13].

The present study has several limitations that should be mentioned. First, the NEISS-AIP does not provide information on specific injuries. For instance, hip fractures are defined as lower trunk injuries. Second, injury severity, comorbidities, or mortality status are not reported in the NEISS-AIP. Finally, older adults admitted directly to the hospital were not included in this analysis [14]. Despite these limitations, a major strength of the present findings is its generalizability to the US older population hospitalized after ED visits for fall-related injuries.

In conclusion, hospitalization rates for fall-related injuries in US older adults increased over the past two decades. These findings were predominantly attributed to a marked increase in the incidence of fall-related internal injuries and TBIs. Notably, downward trends in hospitalization rates in women occurred from 2015 onward. As the population ages, it is expected that hospitalizations with fall-related injuries among older adults continue to increase. The presents findings underscore the importance of developing effective community-based programs of fall prevention.

## Figures and Tables

**Table 1:** Trends in fall-related hospitalization rates among older adults by type of injury, 2001–2021.

	**% (95% CI)**	**AAPC (95% CI)**	***Trend 1***	**APC (95% CI)**	***Trend 2***	**APC (95% CI)**
Women (*n* = 133,214)						
Fracture	64.7 (64.4, 65.0)	1.5 (1.0, 2.2)⁣^∗^	2001–2015	2.8 (2.2, 4.6)⁣^∗^	2015–2021	-1.5 (-3.4, 1.0)
Injury	15.3 (15.0, 15.4)	8.8 (7.6, 10.2)⁣^∗^	2001–2015	12.2 (11.3, 15.4)⁣^∗^	2015–2021	0.4 (-3.8, 5.8)
Contusion/abrasion	9.1 (8.8, 9.2)	0.5 (−1.0, 1.9)	2001–2010	4.8 (1.9, 10.7)⁣^∗^	2010–2021	−2.9 (−7.5, −1.0)⁣^∗^
Strain	1.3 (1.2, 1.4)	−3.2 (−5.3, −1.1)⁣^∗^	2001–2010	7.1 (1.6, 16.3)⁣^∗^	2010–2021	−10.9 (−16.3, 7.5)⁣^∗^
Laceration	3.9 (3.8, 4.1)	2.6 (1.6, 3.9)⁣^∗^	2001–2015	4.5 (3.3, 8.2)⁣^∗^	2015–2021	−1.8 (−5.4, 2.3)
Others^a^	5.7 (5.4, 5.7)	4.1 (3.4, 5.2)⁣^∗^	2001–2007	0.1 (−2.5, 3.7)	2007–2021	5.9 (4.9, 8.7)⁣^∗^
Men (*n* = 69,939)						
Fracture	48.5 (48.0, 48.9)	2.5 (1.9, 3.2)⁣^∗^	—	—	—	—
Internal injury	23.8 (23.4, 24.2)	10.0 (9.1, 11.2)⁣^∗^	2001–2015	13.1 (11.9, 15.6)⁣^∗^	2015–2021	3.0 (−0.0, 7.7)
Contusion/abrasion	12.2 (11.8, 12.4)	2.9 (1.9, 3.8)⁣^∗^	2001–2010	5.8 (3.8, 9.7)⁣^∗^	2010–2021	0.5 (−2.5, 1.9)
Strain	1.3 (1.2, 1.4)	−0.6 (−3.5, 2.3)	2001–2010	8.9 (2.6, 21.6)⁣^∗^	2010–2021	−7.8 (−16.0, −3.8)⁣^∗^
Laceration	6.7 (6.4, 6.9)	3.7 (2.1, 5.2)⁣^∗^	—	—	—	—
Others^a^	7.5 (7.3, 7.8)	4.6 (3.7, 5.9)⁣^∗^	2001–2007	−4.3 (−6.9, 0.9)	2007–2021	8.6 (7.2, 11.2)⁣^∗^

*Note:* AAPC: average annual percent change in age-adjusted rates between 2001 and 2021. APC: annual percent change in age-adjusted rates for a specific period.

^a^Ingestion, aspiration, burn, concussion, amputation, crushing, dislocation, hematoma, dental injury, nerve damage, puncture, anoxia, hemorrhage, poisoning, and avulsion.

⁣^∗^The AAPC or APC is significantly different from zero at alpha = 0.05 level.

**Table 2:** Trends in fall-related injury hospitalization rates among older adults by primary–secondary body part-grouped, 2001–2021.

	**% (95% CI)**	**AAPC (95% CI)**	***Trend 1***	**APC (95% CI)**	***Trend 2***	**APC (95% CI)**
Women (*n* = 133,214)						
Head/neck	26.2 (25.9, 26.5)	5.8 (4.8, 6.9)⁣^∗^	2001–2015	8.4 (7.4, 10.8)⁣^∗^	2015–2021	−0.1 (−3.7, 4.2)
Upper trunk^a^	8.6 (8.1, 8.7)	4.1 (3.5, 4.9)⁣^∗^	2001–2015	5.8 (5.1, 7.7)⁣^∗^	2015–2021	0.3 (−2.1, 3.3)
Lower trunk^b^	42.6 (42.0, 42.7)	−0.1 (−0.7, 0.6)	2001–2015	1.6 (0.9, 3.2)⁣^∗^	2015–2021	−4.0 (−6.0, −1.1)⁣^∗^
Arm/hand	7.7 (7.6, 7.9)	1.7 (1.0, 2.7)⁣^∗^	2001–2015	3.9 (3.0, 5.9)⁣^∗^	2015–2021	−3.0 (−5.5, 0.5)
Leg/foot	14.9 (14.3, 15.0)	3.7 (2.9, 4.6)⁣^∗^	—	—	—	—
Men (*n* = 69,939)						
Head/neck	39.3 (38.5, 39.4)	7.1 (6.4, 8.0)⁣^∗^	2001–2015	9.0 (8.1, 11.0)⁣^∗^	2015–2021	2.7 (0.2, 6.1)⁣^∗^
Upper trunk^a^	10.6 (10.1, 10.7)	5.8 (5.1, 6.4)⁣^∗^	—	—	—	—
Lower trunk^b^	32.4 (31.5, 32.6)	0.3 (−0.3, 1.1)	2001–2015	1.8 (1.1, 3.6)⁣^∗^	2015–2021	−3.1 (−5.3, −0.4)⁣^∗^
Arm/hand	6.3 (6.0, 6.6)	4.3 (3.2, 5.4)⁣^∗^	—	—	—	—
Leg/foot	11.4 (10.7, 11.4)	5.1 (3.5, 6.8)⁣^∗^	—	—	—	—

*Note:* AAPC: average annual percent change in age-adjusted rates between 2001 and 2021. APC: annual percent change in age-adjusted rates for a specific period.

^a^Thoracic spine, ribs, and sternum.

^b^Lumbar spine, pelvis, and hip.

⁣^∗^The AAPC or APC is significantly different from zero at alpha = 0.05 level.

## Data Availability

The data that support the findings of this study are available upon request from the corresponding author.
